# King's College Hospital

**Published:** 1892-05-21

**Authors:** 


### FESTIVAL DINNERS.

### I.—KING'S COLLEGE HOSPITAL.

The festival dinner of King's College Hospital took place on the 14th inst. at the Freemasons' Tavern, the Duke of Fife presiding.

The Chairman, in giving the toast of "Success to King's College Hospital," said that the modern hospital was a great house where all guests were unbidden and free to enter, their only claim being that they were strangers, sick and poor. They usually came from wretched homes, where that comfort and quiet which were associated with the sick room were unknown, and where the strain of their overwrought nerves could not find rest. In days gone by, when hospitals did not exist, the poor, the sick, the infirm, and the insane were huddled together in the common poorhouse, or even left to increase the misery of their squalid homes; but in such a civilisation as ours, with its great industrial population, hospitals became an absolute necessity, and must now be honoured, apart from the good they did to the suffering poor, as schools of medical science and means of developing the highest art of surgery. (Hear, hear.) In fact, hospitals were indispensable to medical education and the diffusion of medical science. King's College Hospital was founded 54 years ago, and was situated in an interesting and historical part of London, abounding in old and picturesque houses, which had known little change, except in occupants, since the days of the Stuarts. He fancied that at no time could they have been very remarkable for their sanitary condition; but, overflowing as they now were with a teeming population, he feared that they constituted a veritable forcing ground for every kind of disease. That noble institution of King's College Hospital, which he had visited with very great interest, stood as a sort of protest amidst its unfavourable surroundings, and held out a helping hand where it was most needed to the sick and the destitute. In going through its wards no one could help feeling struck by the home-like comfort, the high medical skill, and the patient nursing which were everywhere bestowed upon its inmates. That hospital, like every charitable institution in this country, stood in need of financial aid. It had less invested capital than any general hospital of its size in London, and he was there that night to ask them not to allow its good and valuable work to languish for want of funds. The year 1890 was a red-letter day in its existence, because then all its wards were re-opened and were now open; but during the five years previous to that date two had to be closed for want of sufficient funds. He appealed to their generosity not to allow that unfortunate state of things to recur. He gathered from the report that last year the financial position of the hospital was not quite so good as in the previous year. Its assured income was very small—indeed, under £1,000 a year, leaving from £17,000 to £18,000 a year to be raised by subscriptions, donations, and legacies. All who were interested in the hospital work told him that the best and soundest mode of supporting that institution was to increase the list of annual subscribers; but in the case of King's College Hospital, owing to the very large sum which had to be annually raised, donations and legacies were an imperative necessity. He hoped that this year would see the list of annual subscribers very largely developed in addition to a very considerable increase in the number of donations and legacies. (Loud cheers.)

The Archbishop of Canterbury, in responding for the Church, said that Englishmen never felt a stronger blow to be struck at the things they cared most for, though of a different faith in some respects, than they felt when the French Government ordered that the hospitals should no longer be visited by the priests or nursed by religious ladies. Such a thing was unimaginable in our own country. Here we did feel that healing and religion must go hand in hand. (Cheers.) He maintained that King's College Hospital had a great claim upon the nation for the persistency with which it carried on its good work, and with it such noted men and women were engaged in ministering to the wants of the poor, and it would be impossible for such institutions as this hospital to have warmer or more constant supporters than were found among the body on whose behalf he had the pleasure to speak.

Subscriptions and donations amounting to £3,190 were announced.

